# Transgelin gene is frequently downregulated by promoter DNA hypermethylation in breast cancer

**DOI:** 10.1186/s13148-015-0138-5

**Published:** 2015-09-28

**Authors:** Nilufer Sayar, Gurbet Karahan, Ozlen Konu, Betul Bozkurt, Onder Bozdogan, Isik G. Yulug

**Affiliations:** Department of Molecular Biology and Genetics, Bilkent University, Faculty of Science, TR-06800 Ankara, Turkey; Department of General Surgery, Ankara Numune Training and Research Hospital, 06100 Ankara, Turkey; Department of Pathology, Ankara Numune Training and Research Hospital, 06100 Ankara, Turkey

**Keywords:** DNA methylation, Hypermethylation, Breast cancer, TAGLN, SM22 alpha, Prognosis, Diagnosis

## Abstract

**Background:**

CpG hypermethylation in gene promoters is a frequent mechanism of tumor suppressor gene silencing in various types of cancers. It usually occurs at early steps of cancer progression and can be detected easily, giving rise to development of promising biomarkers for both detection and progression of cancer, including breast cancer. 5-aza-2′-deoxycytidine (AZA) is a DNA demethylating and anti-cancer agent resulting in induction of genes suppressed via DNA hypermethylation.

**Results:**

Using microarray expression profiling of AZA- or DMSO-treated breast cancer and non-tumorigenic breast (NTB) cells, we identified for the first time *TAGLN* gene as a target of DNA hypermethylation in breast cancer. *TAGLN* expression was significantly and frequently downregulated via promoter DNA hypermethylation in breast cancer cells compared to NTB cells, and also in 13/21 (61.9 %) of breast tumors compared to matched normal tissues. Analyses of public microarray methylation data showed that *TAGLN* was also hypermethylated in 63.02 % of tumors compared to normal tissues; relapse-free survival of patients was worse with higher *TAGLN* methylation; and methylation levels could discriminate between tumors and healthy tissues with 83.14 % sensitivity and 100 % specificity. Additionally, qRT-PCR and immunohistochemistry experiments showed that *TAGLN* expression was significantly downregulated in two more independent sets of breast tumors compared to normal tissues and was lower in tumors with poor prognosis. Colony formation was increased in *TAGLN* silenced NTB cells, while decreased in overexpressing BC cells.

**Conclusions:**

*TAGLN* gene is frequently downregulated by DNA hypermethylation, and *TAGLN* promoter methylation profiles could serve as a future diagnostic biomarker, with possible clinical impact regarding the prognosis in breast cancer.

**Electronic supplementary material:**

The online version of this article (doi:10.1186/s13148-015-0138-5) contains supplementary material, which is available to authorized users.

## Background

Breast cancer is the most common cancer in women, constituting 29 % of new cancer cases estimated for 2015 in US. Breast cancer can be classified according to molecular and histological subtypes with the most aggressive behavior usually being attributed to the triple negative (TN) subtypes, characterized with loss of estrogen, progesterone, and Her2 receptor expressions [1–3].

Epigenetic changes are heritable changes in gene expression patterns that do not involve alterations in the nucleotide sequence of DNA [4]. DNA methylation is a well-known mechanism for epigenetic gene silencing in mammals. CpG islands are CpG-rich regions around the promoters; 5′ untranslated regions and first exons of many genes and are usually unmethylated in normal cells [5]. The methylated state of a CpG island usually refers to transcriptionally repressed promoters [5]. Tumor suppressor genes (TSG) may become inactivated during tumorigenesis through hypermethylation of associated CpG islands, providing several survival advantages [5–8].

5-aza-2′-deoxycytidine (Decitabine, AZA), a cytosine analogue that causes covalent arrest of DNA methyltransferases (DNMT) upon binding, is one of the most commonly used DNMT inhibitors in cultured cells and to treat cancer patients [9, 10]. Use of demethylating agents together with expression microarrays enables the analysis of genes that may be regulated by hypermethylation of their promoter regions [11]. Discovering these genes is essential not only for therapeutic achievements but can also serve diagnostic, prognostic, and monitoring purposes [12]. Detection of hypermethylated DNA as cancer biomarkers is advantageous regarding the stable nature of DNA, ease of detection, and specificity of DNA hypermethylation for tumor cells [13]. Many known cancer-related genes like *RASSF1A*, *APC*, *BRCA1*, *GSTP1*, and *DAPK1* can be detected from serum or blood of breast cancer patients [14, 15]. Yet, there is still demand for novel diagnosis markers for detection of early breast cancer. In this study, we utilized microarray expression profiling of AZA-treated breast cancer (BC) and non-tumorigenic breast (NTB) cell lines, and identified *TAGLN* gene to be a frequently hypermethylated gene and a potential future biomarker in breast cancer.

Transgelin (TAGLN, SM22α) is an actin-binding protein that is abundantly expressed in smooth muscle cells (SMC) [16–19]. It is downregulated in transformed fibroblasts and several cancer cell lines and tissues [20–24]. *TAGLN* gene function has been associated with increased senescence in fibroblasts [25–27], and with increased apoptosis in glomerular epithelial cells and prostate cancer cells [28, 29]. TAGLN suppresses MMP9 [30], a known element of invasion [31], and therefore prevents the migration of colon and prostate cancer cells [32]. Controversially, it has also been found to be increased in gastric cancer and in lymph node (LN) metastasis of colon cancer [33, 34].

Here, we show that *TAGLN* is an epigenetically suppressed candidate epigenetic biomarker for diagnosis in breast cancer, by presenting its consistent downregulation of expression and frequent hypermethylation in breast carcinoma cell lines as well as in three independent breast tumor tissue cohorts. We also provide evidence that TAGLN decreases the proliferation potentials of BC and NTB cells. Better survival of breast cancer patients with higher expression or lower promoter methylation of *TAGLN*, in addition to competence of its promoter methylation levels to discriminate cancer from healthy tissue, emphasize a possible clinical impact for this gene in breast cancer.

## Results

### Changes in the transcriptome of breast cell lines upon AZA treatment

**Fig. 1:**
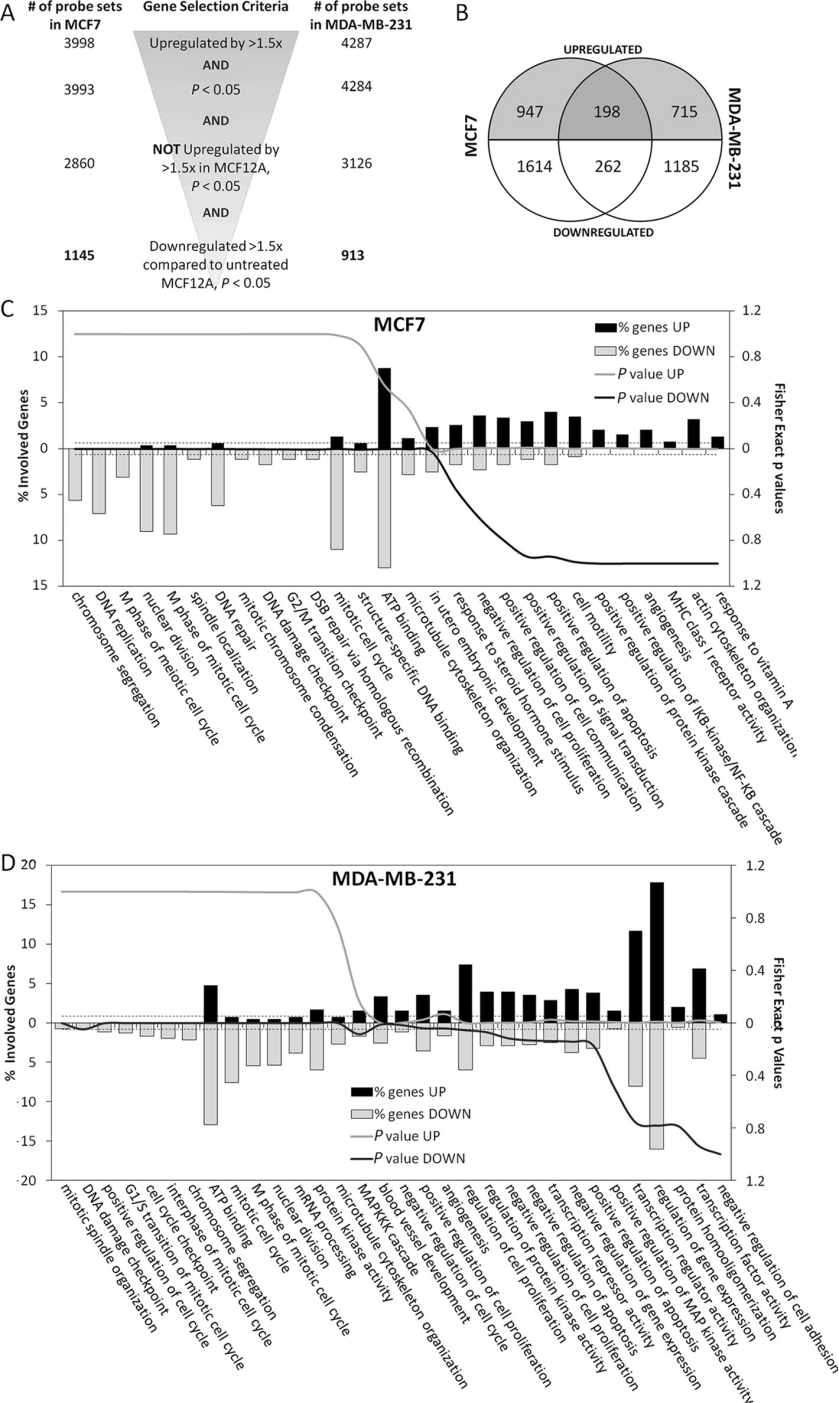
Analyses of AZA affected genes in BC cell lines. **a** The outline of the gene selection process. The numbers on the *left* and *right* represent the probe sets obtained after the filtering process for gene selection in MCF7 and MDA-MB-231 cell lines, respectively. **b** Number of probe sets significantly up/downregulated by AZA treatment, determined by the selection process given in (**a**). **c**, **d** DAVID analyses of Pathways affected upon AZA treatment in MCF7 cells (**c**) and MDA-MB 231 cells (**d**). *Bars* represent the percentage of up- and downregulated genes involved in the indicated pathways while the lines represent the *P* values for enrichment. *Dashed lines* indicate *P* = 0.05 thresholds

We treated the MCF7 and MDA-MB-231 BC cells and a NTB cell line MCF12A with AZA or corresponding DMSO controls before subjecting them to microarray analysis to identify new TSG targets for promoter DNA hypermethylation in breast cancer. Quality control analyses proved our data to be of high quality, and replicates of each experiment correlated well with each other (data not shown). Expressions of large groups of genes were altered upon AZA treatment by 1.5-fold and above, even at very stringent significance thresholds (Additional file 1 and Additional file 4: Table S1). A selection procedure was applied to identify the genes that are altered upon AZA treatment in MCF7 and MDA-MB-231 cells, but not in MCF 12A cells (Additional file 2 and Fig. 1a**)**. Gene lists, generated using this procedure, were used for further analyses (Additional file 3). Probe sets commonly or differentially altered in MCF7 and MDA-MB-231 cell lines were also determined (Fig. 1b). We found that 17 and 22 % of the probe sets induced in MCF7 or MDA-MB-231 cells, respectively, were induced in common while the remaining genes were cell-line specific.

Functional annotation and clustering of significantly altered genes in either the MCF7 or MDA-MB-231 cell lines were analyzed using the DAVID Functional Analysis Tool (Fig. 1c, d) [35, 36]. Pathways mainly involved in mitosis and cell cycle were significantly enriched among the downregulated genes in both of the cell lines. Pathways related to cell communication and motility, signal transduction, cell proliferation, and apoptosis regulation were significantly enriched among the AZA-induced genes in MCF7 (Fig. 1c) while regulation of gene expression was highlighted in addition to these pathways in MDA-MB-231 cells (Fig. 1d).

Paired *t* tests between seven AZA-treated and untreated BC cell lines of a publicly available independent dataset (GSE20713 [37]) revealed that 53.5 % of the probe sets that were significantly upregulated upon AZA treatment in both MCF7 and MDA-MB-231 cells in our experimental setup were also significantly upregulated in the AZA-treated BC cell lines of this independent study (*P* < 0.05, Additional file 4: Table S2).

Probe set lists were screened for the presence of CpG islands in the proximity of their promoter regions, and the induction in expression levels of several genes were validated by qRT-PCR among the genes with proximal CpG islands. *TAGLN* emerged as a strong candidate gene among a large number of genes, as it was commonly and significantly upregulated upon AZA treatment in both MCF7 (by 27.3-fold) and MDA-MB-231 (by 1.77-fold) cell lines (Additional file 3), and our bioinformatics analyses showed that *TAGLN* expression was significantly downregulated (>2-fold, *P* < 0.05) in breast tumor tissues of 8 out of 13 (62 %) breast cancer datasets included in Oncomine database [38] (Additional file 4: Table S3). Moreover, *TAGLN* promoter region was previously shown to be responsive to hypermethylation in smooth muscle cells [39]. Based on these findings, we selected this gene for further analyses.

### *TAGLN* gene expression and promoter methylation analyses in breast carcinoma cell lines

**Fig. 2:**
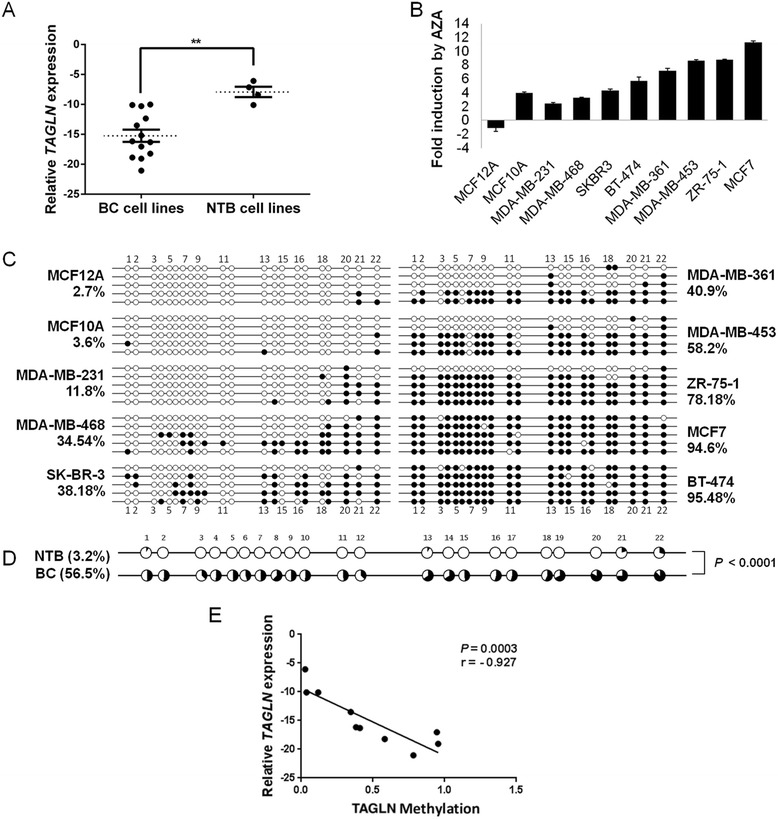
Expression and promoter methylation analyses of *TAGLN* gene in BC cell lines. **a** Box-plot analysis based on qRT-PCR results showing strong downregulation of *TAGLN* expression in BC cell lines compared to NTB cell lines. Log_2_ expression levels relative to *GAPDH* reference gene are shown. *Horizontal dashed lines* represent median values. *Error bars*: standard error of means (SEM). **P < 0.01; Mann–Whitney Test. **b** qRT-PCR analysis of AZA-treated BC and NTB cell lines indicating high levels of induction in *TAGLN* expression levels. Values represent the log_2_ ratios of expression levels in AZA-treated cell lines to those in their DMSO-treated cells. **c** QUMA software analyses based on bisulfite sequencing of BC and NTB cell lines showing hypermethylation in *TAGLN* promoter in BC cells. Percent values represent the average methylation of 5 clones for each cell line. *Full circles*: methylated; *Empty circles*: unmethylated. Sequence numbers of CpGs are shown *above circles*. **d** QUMA analysis showing significantly higher methylation in BC cells compared to NTB; Mann–Whitney test. Methylated DNA ratio: *black area*; unmethylated ratio: *white area* in the pie chart. **e** Scatter plot showing the spearman correlation of *TAGLN* methylation status with log_2_(expression) levels in BC and NTB cell lines

*TAGLN* gene expression was analyzed with qRT-PCR and found to be significantly downregulated in 15 different breast carcinoma cell lines compared to the non-tumorigenic cell lines (*P* = 0.0034, Fig. 2a**)**. The expression of *TAGLN* was restored in all analyzed cell lines upon AZA treatment, except for NTB cell line MCF12A (Fig. 2b**)**. Expression levels of *TAGLN* were concordant in microarray and qRT-PCR experiments, when fold induction levels upon AZA treatment were compared in MCF7, MDA-MB-231, and MCF12A cell lines.

Bisulfite sequencing of *TAGLN* promoter region was used to characterize *TAGLN* methylation status in eight BC and two NTB cell lines (Fig. 2c). The analyzed promoter region was significantly hypermethylated in majority of BC cell lines (with an average of 56.5 % methylation) compared to the NTB cell lines (with an average of 3.2 % methylation; *P <* 0.0001, Fig. 2d). Moreover, mRNA expression and methylation levels of *TAGLN* were significantly and negatively correlated (Spearman *r* = −0.927, *P* = 0.0003, Fig. 2e) suggesting that the expression of *TAGLN* was downregulated by promoter DNA methylation in BC cell lines.

### *TAGLN* is downregulated by promoter hypermethylation in human breast tumor tissues

**Fig. 3:**
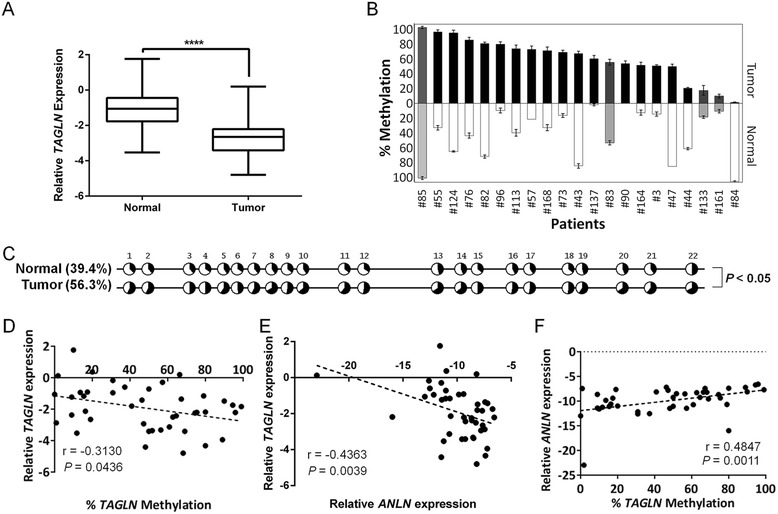
Expression and promoter methylation analyses of *TAGLN* gene in normal matched tumor tissues. **a** Box-plot analysis showing strong downregulation in average *TAGLN* expression in tumor tissues compared to matched normal tissues. Log_2_ expression levels relative to geometric means of *ACTB* and *SDHA* reference genes were determined by qRT-PCR. ****P < 0.0001, Wilcoxon signed rank test. **b** Comparative bar charts of bisulfite sequencing based percent methylation scores of matched tumor and normal pairs showing significance hypermethylation in majority of tumors. Methylation percentages represent the average of five clones for each tissue. *Black-white bar* pairs represent significant (*P* < 0.05) differences between tumor and normal tissues (Wilcoxon signed rank test). *Error bars* represent SEM values. **c** Overall average methylation levels based on QUMA were significantly higher in breast tumors compared to normal tissues; Wilcoxon signed rank test. Methylated DNA ratio; *black area* and unmethylated; *white area* in the pie chart. **d–f** Scatter plots show correlations between relative *TAGLN* expression levels and methylation status of *TAGLN* promoter in tumor and normal tissues; Spearman *r* = −0.3130, *P* = 0.0436 **d**; relative *TAGLN* and *ANLN* expressions; Spearman *r* = −0.4363; *P* = 0.0039 **e**; and relative *ANLN* expression and *TAGLN* methylation; Spearman *r* = 0.4847, *P* = 0.0011 **f**. All expression values are log_2_. The correlations were robust to outliers

To profile the expression and methylation status of *TAGLN* in breast tumor tissues, *TAGLN* mRNA expression and promoter methylation levels were determined in 21 breast tumor samples and their pair-matched normal tissues (Table 1). The expression of *TAGLN* was found to be significantly downregulated in tumors compared to matched normal breast tissues by qRT-PCR (19/21 tumors, 90.5 %, *P <* 0.0001, Fig. 3a). The average relative expression (ARE) ± standard error of means (SEM) was 0.476 ± 0.1520 for normal tissues while it was 0.146 ± 0.0511 for tumor tissues, with an average decrease in *TAGLN* expression of 3.255-fold in breast tumor tissues.

**Table 1:** Pathological features of normal matched breast tumor tissues

Patient code	Lymph node	Grade	Stage	ER status	PR status	Her2 status
#003	1	1	N/A	1	1	0
#043	0	2	N/A	1	1	1
#044	1	2	2B	0	1	1
#047	1	2	4	1	0	1
#055	0	2	N/A	1	1	1
#057	0	N/A	2B	0	0	0
#073	1	2	N/A	1	0	1
#076	1	3	2A	0	0	1
#082	0	1	2B	1	0	1
#083	1	2	2B	1	0	1
#084	1	3	N/A	0	0	0
#085	0	2	2A	0	1	1
#090	1	2	2B	1	1	1
#096	0	1	2A	0	1	0
#113	1	1	3B	1	0	1
#124	1	1	2A	1	0	1
#137	1	N/A	3A	1	1	N/A
#133	N/A	N/A	N/A	N/A	N/A	N/A
#161	1	2	2A	1	0	0
#164	0	2	2B	1	1	0
#168	1	2	2B	0	1	1

*ER* estrogen receptor, *PR* progesterone receptor, *Her2* Erbb2 receptor amplification, *N/A* information regarding the pathological feature is missing, *1* positive for the given condition, *0* negative for the given condition

The promoter methylation status of *TAGLN* was analyzed via the bisulfite sequencing method, and the examined region was found to be significantly hypermethylated in 13 (61.9 %) tumors (*P <* 0.05, Fig. 3b, Table 2) compared to matched normal tissues; hypermethylation was consistent across all grades (Table 2). The overall methylation levels were also significantly higher in tumor tissues in comparison with normals (*P* = 0.0263, Fig. 3c). *TAGLN* mRNA expression and the promoter methylation ratios were significantly and negatively correlated in breast tissues (Spearman *r* = −0.31, *P* = 0.044, Fig. 3d) as in BC cell lines, suggesting that *TAGLN* transcription could be regulated by DNA methylation. We used *ANLN* (Anillin) gene expression, a poor prognosis and high cellular proliferation marker in breast cancer patients [40], to better infer proliferative capacity and prognostic status of the tumors. Expression of *TAGLN* was negatively (Spearman *r* = −0.44, *P* = 0.0039) and the promoter methylation of *TAGLN* was positively (Spearman *r* = 0.48, *P* = 0.0011) correlated with mRNA expression of *ANLN* gene (Fig. 3e, f). This shows that *TAGLN* is likely to be downregulated more by promoter DNA hypermethylation in breast tumors with worse prognosis. When the tumors’ pathological features were analyzed, promoter methylation, but not expression, of *TAGLN* was significantly decreased in LN negative tumors when compared with LN positives (*P* = 0.0476, Additional file 5: Figure S1A-B) while both expression and methylation levels did not exhibit any significant difference between grades, stages, and hormone statuses in our tumor-normal matched breast tissue panel.

**Table 2:** Frequency of *TAGLN* hypermethylation in tumor and normal breast tissues

Study	Analyzed CpGs	Cohort	Hypermethylated^c^	Hypomethylated^d^
Matched tumor tissue panel (bisulfite sequencing)^a^	−290 to +117	All patients (*n* = 21)	13 (61.90 %)	4 (19.05 %)
	22 CpGs			
		Grade 1 (*n* = 5)	5 (100.00 %)	0 (0.00 %)
		Grade 2 (*n* = 11)	6 (54.55 %)	3 (27.27 %)
		Grade 3 (*n* = 2)	1 (50.00 %)	1 (50.00 %)
GSE20713 Methylation data (microarray analysis)^b^	−571 and 455	All patients (*n* = 338)	213 (63.02 %)	3 (0.89 %)
	2 CpGs			
		Grade 1 (*n* = 64)	41 (64.06 %)	0 (0.00 %)
		Grade 2 (*n* = 57)	35 (61.40 %)	1 (1.75 %)
		Grade 3 (*n* = 215)	135 (62.79 %)	2 (0.01 %)

^a^Number of tumors were calculated based on average methylation ratio of 22 CpGs in our normal matched tumor tissues

^b^Number of tumors were calculated based on average methylation scores of two probes corresponding to two CpGs at given locations

^c^Number of tumors that are significantly hypermethylated in normal matched tumor tissue panel (*P* < 0.05, Wilcoxon signed rank test). In microarray methylation data, tumors with normalized average methylation score (NAMS) >30 higher than the average normal tissue NAMS were considered hypermethylated

^d^Number of tumors that are significantly hypomethylated in normal matched tumor tissue panel (*P* < 0.05, Wilcoxon signed rank test). In microarray methylation data, tumors with NAMS >30 lower than the average normal tissue NAMS were considered hypomethylated

**Fig. 4:**
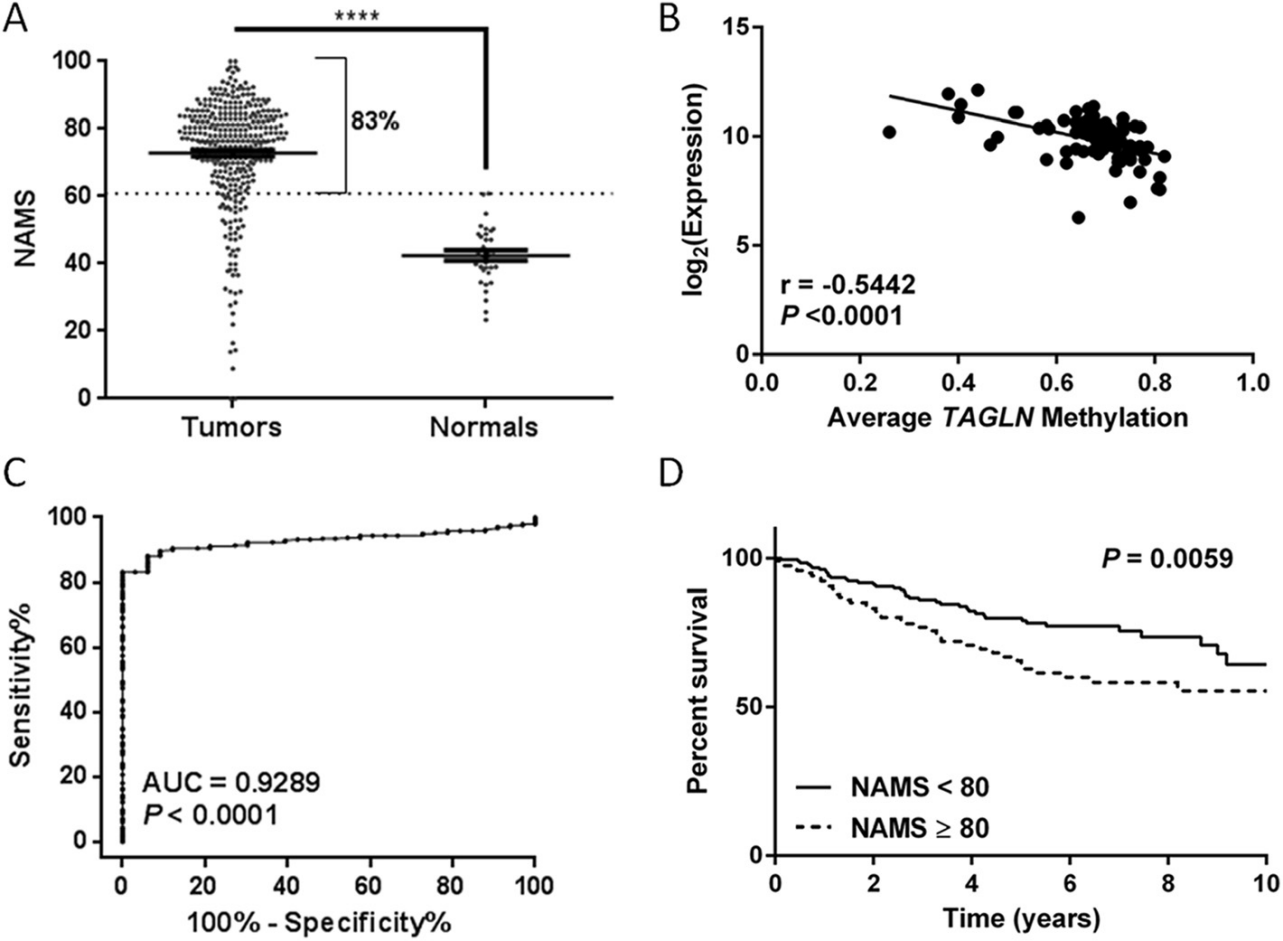
Methylation status of *TAGLN* in public datasets GSE20713 and GSE31979. **a** Graphs showing higher normalized average methylation scores (NAMS) for two probes of *TAGLN* in tumor tissues Mann–Whitney test, ****P < 0.0001. *Horizontal lines*: mean ± SEM. **b** Scatter plot showing correlation of average methylation scores of *TAGLN* probes (GSE20713) to average expression (log_2_) of *TAGLN* probe sets (GSE20711); Spearman *r* = −0.5442, *P* < 0.0001. **c** ROC analyses for discrimination between healthy individuals and breast cancer patients, using *TAGLN* NAMSs. **d** Kaplan-Meier (KM) survival analysis showing higher RFS percentages for patients with tumor *TAGLN* NAMSs ≤80 (best cutoff among tested)

To increase our sample size and extend our DNA methylation analyses to a larger panel with clinical and long-term outcome data, we downloaded the publicly available methylation data of two independent microarray datasets (Super-series: GSE201713 [37], *n* = 247 and GSE31979 [41], *n* = 124), whose distributions for methylation scores were similar, and used the normalized average methylation scores (NAMS) of two probes corresponding to *TAGLN* gene. In support of our results, *TAGLN* probes were significantly hypermethylated in breast tumors (NAMS ± SEM = 72.88 ± 0.9137, *n* = 338) compared to normal tissues (NAMS ± SEM = 42.49 ± 1.541, *n* = 33, *P* < 0.0001, Fig. 4a), even though the CpG dinucleotides corresponding to the probes were not covered by our bisulfite primers. 83 % of the tumors displayed higher methylation scores than the highest score observed in the normal cohort. The average difference between the methylation scores of the tumor and normal tissues was 30.38; thus, the tumors with methylation scores that were >30 higher than the average value for normal tissues were considered as hypermethylated. Accordingly, 63.02 % of the tumors displayed hypermethylation of *TAGLN* probes in this data set (Table 2). When grouped into tumor grades, these ratios were at least 61.40 % for each group (Table 2). Available expression data (GSE20711; *n* = 90) of the same patients were significantly and negatively correlated with *TAGLN* methylation scores (Spearman *r* = −0.5442, *P* < 0.0001, Fig. 4b). This correlation was preserved in each grade and pathological subtype tested except for Luminal B (LumB) tumors, which are known for their hypermethylator phenotypes [42] (data not shown). Receiver-operator characteristics (ROC) analyses of *TAGLN* promoter methylation showed that a threshold of NAMS >60.94, which was derived from the ROC analysis itself, could discriminate healthy individuals from breast cancer patients, with 83.14 % sensitivity and 100 % specificity (area under the curve (AUC) = 0.9289, and 95 % confidence interval (CI): 0.9027 to 0.9552, *P <* 0.0001, Fig. 4c). Moreover, 10-year relapse-free survival (RFS) of patients was significantly decreased with higher tumor methylation of the *TAGLN* probes compared to the patients with lower levels of methylation (*P* = 0.0059, Fig. 4d). NAMSs of the *TAGLN* probes were compared for different pathological features of breast cancer, and *TAGLN* methylation states were similar, independent of grade, Her2, and ER states, and sizes of the tumors.

### *TAGLN* mRNA and protein is downregulated in independent sets of breast tumor tissues

**Fig. 5:**
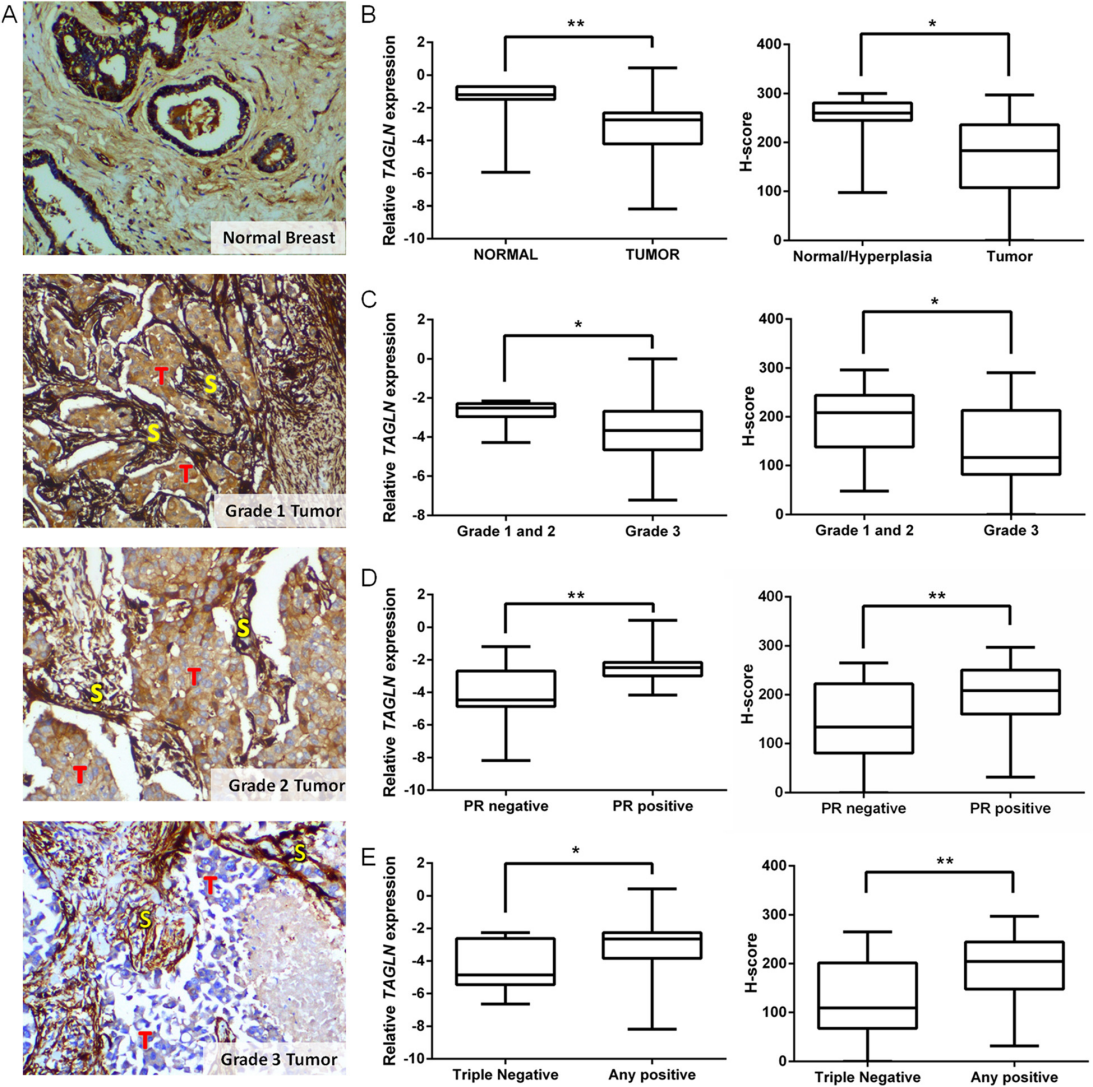
*TAGLN* expression in independent sets of tumor and normal breast tissues. **a** Examples of IHC staining of TAGLN in normal and tumor tissues. T (*red*), tumor tissue; S (*yellow*), stroma, ×100 magnification. **b–e** Box-plot analyses showing significant downregulation of *TAGLN* mRNA (*left*) and protein (*right*) expression levels in tumors compared to normal breast tissues (**b**); in grade 3 compared to grade 1 and 2 tumors (**c**); progesterone receptor (PR) negative compared to positive subtypes (**d**); and in triple negative compared to any hormone receptor positive (ER and/or PR and/or Her2 positive, Any positive) tumors (**e**). *Horizontal lines*: maximum, median, and minimum values. *Left panels*: qRT-PCR analysis of *TAGLN* mRNA expression levels (log_2_ transformed) in a cDNA array (*n* = 7 normal, *n* = 41 tumor tissues) relative to geometric means of *ACTB* and *SDHA* mRNA reference genes. *Right panels*: IHC staining of TAGLN protein in a tissue array (*n* = 67 tumor, *n* = 7 normal tissues). H-score method was used for staining intensity calculation. *P < 0.05, **P < 0.01, Mann–Whitney Test

To confirm downregulation of *TAGLN* in both protein and mRNA levels in other independent breast tumor tissue panels of larger sample sizes with pathological information, expression of *TAGLN* was examined in a breast cancer tissue array by IHC staining (BioChain, Cat.No:Z7020005), and in an independent cDNA panel by qRT-PCR (OriGene, Cat.No:BCRT101). Both protein (*P* = 0.0116) and mRNA expression levels (*P* = 0.0072) of *TAGLN* were significantly downregulated in breast tumors in comparison to non-tumor tissues (Fig. 5a, b). Average of relative mRNA expression, ARE_(mRNA)_ ± SEM was 0.4248 ± 0.0792 in normal tissues (*n* = 7) and 0.1858 ± 0.0387 in tumor tissues (*n* = 41). Average histochemical score H-score ± SEM was 243.6 ± 25.35 in normal tissues (*n* = 7), with extensive staining in myoepithelial cells (Fig. 5a), and was 169.8 ± 9.773 in tumor tissues (*n* = 67). In both the tissue and cDNA arrays, *TAGLN* expression was downregulated in grade 3 tumors when compared to grade 1 and 2 tumors (*P* = 0.049 and *P* = 0.0329, respectively, Fig. 5c), upregulated in progesterone receptor PR (+) tumors (*P* = 0.0031 and *P* = 0.0043, respectively, Fig. 5d) and in any hormone receptor positive tumors with respect to triple negative tumor types (*P* = 0.0063 and *P* = 0.0489, respectively, Fig. 5e). *TAGLN* expression levels were not significantly different in estrogen receptor (ER) or human epidermal growth factor receptor 2 (Her2) positive or negative tumors or in different stage groups.

The tissue panels used in this study did not have enough information about patient survival data. KM plotter [43], which uses public microarray expression data for survival analyses, was used to test the effect of *TAGLN* expression on OS or RFS of breast cancer patients (Additional file 5: Figure S2). There was no significant difference between either OS or RFS of the patients based on their tumor expression levels of *TAGLN* when all patient cohorts were analyzed (Additional file 5: Figure S2A). However, when the patients were grouped for tumor grades, higher expression of *TAGLN* was associated with increased OS and RFS probabilities in patients with grade 1 and 2 tumors (Additional file 5: Figure S2B). In grade 3 tumors, the differences were not significant.

### TAGLN decreases colony formation potentials of breast carcinoma cell lines

**Fig. 6:**
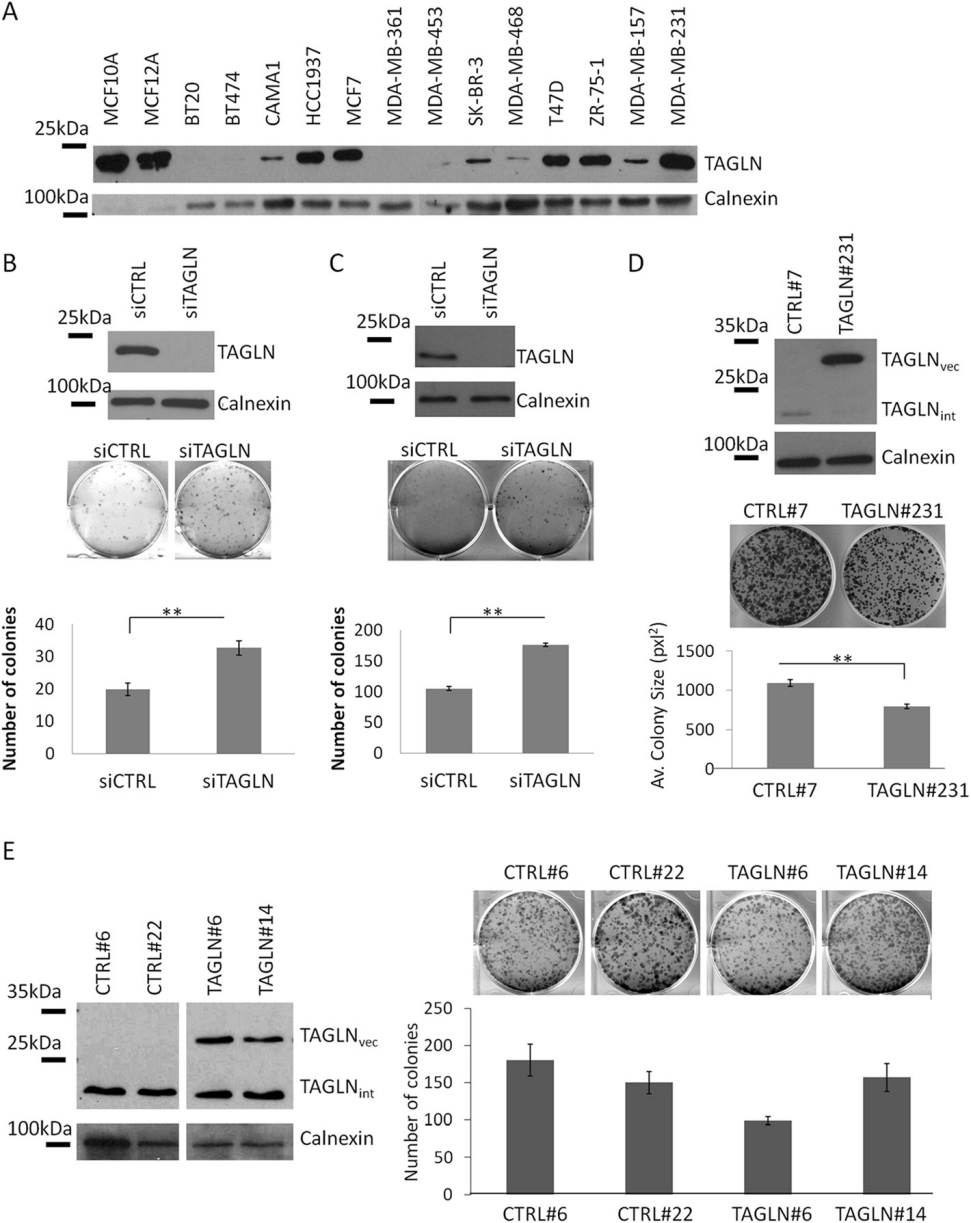
Effect of *TAGLN* overexpression and silencing on breast cell lines. **a** Western blot analysis of TAGLN in breast carcinoma and non-tumorigenic breast cell lines. **b–c** Western blot shows *TAGLN* silencing in MCF10A (**b**) and MCF12A (**c**) cells (*upper panels*). 2D colony formation assays (*middle* and *lower panels*) show increased colony formation in *TAGLN* silenced (si*TAGLN*) MCF10A (**b**) and MCF12A (**c**) cells compared to controls (siCTRL). **d**–**e** Western blot analyses of *TAGLN* overexpression and subsequent colony formation assays in MDA-MB-361 (**d**, *upper panel*), and MDA-MB-157 (**e**, *left panel*) cell clones. Overexpression of ectopic TAGLN (TAGLN_vec)_ and levels of endogenous TAGLN (TAGLN_int)_ were determined with TAGLN specific antibody in clones. *TAGLN* overexpression caused higher cell proliferation in MDA-MB-361 cells (**e**), while did not affect MDA-MB-157 cells (**f**). Each assay was conducted at least in quadruplicates. Two sample *t* test was used for comparisons. *Error bars* represent standard errors. **P < 0.01

Since *TAGLN* was frequently hypermethylated and consistently downregulated in both the BC cell lines and three independent panels of tumor tissues, we analyzed the functional role of TAGLN in BC and NTB cells. *TAGLN* gene was silenced in MCF10A and MCF12A cells with abundant expression (Fig. 6a–c), and was overexpressed it in MDA-MB-361 and MDA-MB-157 BC cell lines (Fig. 6d, e) that expressed *TAGLN* at zero to intermediate levels, respectively (Fig. 6a). 2D colony formation assays were performed to test the effect of TAGLN on proliferation potentials of these cells. Compared to control siRNA (siCTRL), si*TAGLN* transfected MCF10A and MCF12A cells formed significantly (*P* = 0.0013 and *P* = 0.0016, respectively) higher number of colonies (Fig. 6b, c, middle and lower panels). Colony formation capabilities of MDA-MB-361 cells, but not MDA-MB-157 cells, were significantly impaired when they overexpressed TAGLN protein (Fig. 6d, middle and lower panels and Fig. 6e, right panel). To examine possible effects of TAGLN on migration and invasion potentials of BC cells, we performed *in vitro* wound healing and Matrigel invasion assays in normally motile MDA-MB-157 cells; however, *TAGLN* overexpression in these cells did not affect either wound closure or Matrigel invasion capacities of these cells (Additional file 5: Figure S3).

## Discussion

The combination of high-throughput expression profiling technology and treatment of cells with various DNA methylation inhibitors has been widely used to discover genome-wide effects of these drugs or to identify specific genes involved in the related pathways. Here, we used the same technology and identified *TAGLN* as an important candidate biomarker, frequently downregulated by promoter hypermethylation in breast cancer. A recent study (GEO ID: GSE22250) also used this method in breast cancer [37], in which multiple breast cancer cell lines without biological replicates were exposed to 1 μM AZA. About half of the probe sets commonly upregulated in BC cells by AZA treatment in our setup were also upregulated (*P <* 0.05) in the AZA-treated BC cell lines in the aforementioned study, although they used a lower dose (1 μM) of AZA. This indicates a potential dose dependency of AZA treatment such that some of the genes may require higher concentration of AZA to be induced. However, we determined *TAGLN* to be upregulated by 2.38-fold (average of two probe sets, *P* < 0.05, paired *t* test) also in GSE22250 study, yet this was not reported by the authors.

The pathway analyses of 5 μM AZA-induced genes revealed that the most significantly enriched pathways in MCF7 cells were the cell motility, angiogenesis, cytoskeleton, and cell communication pathways, which could have been suppressed in these non-motile cells under normal conditions by DNA methylation. Yet, motility-associated genes in invasive and motile MDA-MB-231 cells might have already been active even before the AZA treatment, so only gene expression and protein kinase regulation pathways were enriched upon AZA treatment. Commonly induced pathways in both cell lines were those relatively known as characteristics of the AZA effect on cancer cell lines [44–47], while commonly downregulated pathways mostly consisted of the cell cycle, cell division and metabolism-related pathways, potentially resulting in cessation of cell growth and proliferation when deactivated, enabling AZA’s anti-cancer effect. Yet, most of these downregulated genes could be possibly repressed due to secondary effects of AZA treatment, as it caused induction of several transcription regulatory genes.

The promoter region of *TAGLN*, one of the strongest candidate genes among many probe sets, was previously found to be methylated in hepatocellular carcinoma and normal hepatocytes [48] and was hypermethylated in colorectal carcinoma [49]. Using a luciferase reporter gene construct containing the 5′-flanking region of *TAGLN* promoter, and *in vitro* methylation of the CpG dinucleotides within this region, Yamamura et al*.* [39] showed that the transcriptional activity of *TAGLN* promoter could indeed be controlled by DNA methylation in smooth muscle cells. However, transcriptional regulation of this gene in breast cancer has never been studied before.

Our study is the first to document the regulation of *TAGLN* gene expression by promoter hypermethylation in both breast carcinoma cell lines and normal matched tumor breast tissues, as well as in tumor tissues of large set of publicly available data by means of bioinformatics analyses. Frequent hypermethylation of the *TAGLN* promoter region, in breast cancer even at low-grade tumors, is comparable to the hypermethylation frequencies of known TSGs in breast and other types of tumors [50]. High frequency of DNA hypermethylation of certain genes enables detection of circulating cell-free tumor DNA from easily accessible and noninvasive sites, like serum or plasma, of cancer patients, with quick and sensitive techniques such as methylation-specific PCR (MSP) [14, 15, 51]. Even though it is possible to detect and analyze hypermethylated genes from the fine needle aspirates, nipple aspirates, and ductal lavage for breast cancer patients [52–54], diagnosis from serum or plasma is more patient friendly and noninvasive and can be easily repeated during the follow-up period. Thus, pursuit for blood-based tests for diagnosis of breast cancer has been an ever expanding research area [12, 55]. The most common biomarker candidates for detection of breast cancer from serum is *RASSF1A* gene, with 100 % specificity and 75 % sensitivity [56], and *RARβ* with 94 % specificity and 87 % sensitivity [57]. Based on the high frequency of hypermethylation in breast cancer patients compared to healthy tissues, *TAGLN* promoter methylation levels can be used as an epigenetic based biomarker for diagnosis, either alone or together with other frequent markers. Indeed, the results of our ROC analyses with high sensitivity and specificity values, point out *TAGLN* gene methylation as a good candidate for a tumor marker, with 83.14 % sensitivity and 100 % specificity. These ratios are similar to the ones obtained by combining the scores of more than one gene for detection of breast cancer (reviewed in Kristiansen 2013 [12]), and could increase their competence when included in these panels. The power of *TAGLN* promoter hypermethylation as an epigenetic diagnosis biomarker seems promising and should be validated for detection of tumor DNA from serum, plasma, or nipple and ductal fluids in a large, prospective screening study.

*TAGLN* methylation states also relate to the prognosis of breast cancer, as *TAGLN* promoter methylation levels were negatively correlated with cellular proliferation and worse prognosis marker *ANLN* expression in our tumor-normal matched tissue panel [40]. Moreover, the probability of 10-year RFS of patients was greater with lower *TAGLN* methylation scores, independent of grade, stage, and other prognostic factors. These outcomes indicate that hypermethylation of *TAGLN* promoter can also be used as a prognostic marker. Larger cohorts of patients with matched breast cancer and adjacent normal tissues, and available survival information should be used to validate these findings with bisulfite sequencing or other sensitive techniques like MSP.

*TAGLN* expression was previously found to be downregulated in several types of cancer and transformed cells [18, 20–22, 24]. However, studies on breast cancer were limited to a small number of cell lines and tissues, all of which were ductal carcinoma in situ. In this study, we showed strong and consistent downregulation of *TAGLN* mRNA and protein expression in three independent sets of breast tumor tissues compared to non-tumor tissues (total number of samples *n* = 130 tumors and *n* = 35 normal tissues). Furthermore, this is the first time an association between *TAGLN* expression and breast cancer prognosis is suggested by independent findings: (1) negative correlation of *TAGLN* expression with poor prognosis marker *ANLN* [40]; (2) decreased *TAGLN* expression particularly in triple negative breast cancer in addition to all molecular subtypes of breast cancer tissues; and (3) longer 10-year OS and RFS of low-grade breast cancer patients with higher *TAGLN* expression.

TAGLN is usually known as a mesenchymal protein [19], whose expression increases with epithelial to mesenchymal transition (EMT) [58–60]. In this study, we found that the expression of *TAGLN* was higher in grade 1 and 2 tumors compared to grade 3, suggesting that *TAGLN* expression could be decreased even more when the tumor progresses into a more undifferentiated state in breast cancer. This suggests that downregulation of *TAGLN* expression may not be related to the EMT state of breast tumors while its downregulation can be an important marker in breast tumor progression. Future studies with higher numbers of grade 2 and grade 3 tumor samples will help increase confidence in this regard.

Previous data on association of *TAGLN* with metastatic states of other tumor types are also controversial. While some studies negatively correlated *TAGLN* expression with lymph node status in colon [32] and prostate cancers [61], others found positive correlation in colorectal cancer [34]. Increased expression of *TAGLN* in metastatic nerve sheath tumors was also shown [62]. At least part of the discrepancies in the expression levels of *TAGLN* in different studies has been explained as a result of higher *TAGLN* expression in the tumor stroma of invasive tumors rather than the tumor tissue itself, which is revealed by subsequent IHC staining analyses [33, 63–65]. Accordingly, extensive staining of TAGLN in the tumor stroma was also found in our panel of breast tissues stained by IHC, while *TAGLN* gene expression was specifically downregulated in tumor tissues of all grades and pathological types, when compared to normal tissues**.**

While some studies suggest a TSG function for *TAGLN* due to downregulation in different types of tumors [22, 24, 32, 49, 60, 66, 67], others define tumor-promoting activities or overexpression in cancer [34, 68, 69]. Despite these contradictory functions suggested in different types of tumors, our findings support a possible tumor suppressive role of this protein in breast cancer. The observed anti-proliferative effect exerted by TAGLN on breast cells in our study can be caused by its close association with the actin cytoskeleton [17, 70], which has essential roles in several pathways in a cell (reviewed in Hall, 2009 [71]). *TAGLN* expression is known to directly affect actin cytoskeleton dynamics [60, 70, 72, 73]. In yeast, higher expression of *TAGLN* homolog Scp1 decreases actin dynamics, leading to increased permeability of mitochondria and release of reactive oxygen species (ROS), which in turn results in cell death [74]. Similar results have been reported in melanoma cells [75]. Likewise, *TAGLN* depletion in fibroblasts resulted in disrupted actin organization and lower levels of ROS [60]. Thus, lower *TAGLN* expression observed in breast tumors and cell lines can support tumor development or cell proliferation by means of decreased ROS production resulting in longevity, or other mechanisms and pathways involved, which should be further tested in a comprehensive and focused functional study.

TAGLN is known to be involved in SMC migration via formation of podosomes and focal adhesions [73, 76, 77], as well as in lung fibrosis [78]. Controversially, its overexpression results in decreased cellular migration in colon cancer [32, 79]. We found that overexpression of *TAGLN* in MDA-MB-157 cells did not cause any significant change in both the migration and invasion capabilities of these BC cells. It is possible that TAGLN acts differently in mesenchymal SMCs and in epithelial origin of cancer cells, as protein profiles might differ among different cell types, affecting the functions of individual proteins. Further studies are required to unravel possible protein partners of TAGLN in cells with different origins to explain the contradictory outcomes observed in different studies.

## Conclusions

Our novel findings support the essential role of *TAGLN* gene in breast cancer pathogenesis, regarding its negative effect on cellular proliferation and its consistent downregulation in tumors. Based on substantially high methylation frequencies in breast cancer, further studies focusing on *TAGLN* promoter methylation as a diagnostic marker, combined with other biomarkers, should seriously be considered. These further studies with expanded breast cancer patient cohorts and bisulfite sequencing accompanied with expression analyses will determine whether *TAGLN* expression and/or methylation could also be used as a prognostic marker as well.

## Methods

### Cell culture and AZA treatment of breast cell lines

MCF7, MDA-MB-231, MDA-MB-453, MDA-MB-468, BT-20, BT-474, ZR-75-1, HCC1937, MDA-MB-361, MDA-MB-157, and MCF12A cell lines were purchased from American Type Culture Collection (ATCC). MCF10A cell line was kindly provided by Elif Erson Bensan, (METU, Ankara, Turkey). All cell lines were grown as recommended by ATCC. AZA (5 μM, Sigma-Aldrich, A3656) dissolved in DMSO (Applichem, A1584.0100) was used to treat cells for 96 h, and equivalent amount of DMSO was used as control treatment. Media was changed at every 24th hour, and cells were harvested at 96^th^ hour.

### RNA isolation, determination of RNA quality and hybridization to microchips

RNA was isolated from the cell lines with Nucleospin RNA kit (Macherey-Nagel (MN), 740955.5), quantified with NanoDrop ND-1000 spectrophotometer (Thermo Scientific, USA) and the RNA integrity was determined with Agilent 2100 Bioanalyzer (Agilent Technologies, Germany) and all RNA samples had RNA integrity number (RIN) between 9.8–10. Hybridization of the processed RNA samples to the gene chips (HGU-133-Plus 2.0, Affymetrix, USA) were done following the standard Affymetrix protocol, in the Microarray Hybridization Facility, at Bilkent University (Ankara, Turkey).

### Quality control and analysis of the microarray expression data

Data were scanned from the microchips with GCOS software (Affymetrix, USA). Raw data were normalized with justRMA method using BRB-ArrayTools 3.8.0-beta, developed by Dr. Richard Simon and the BRB-ArrayTools Development Team (NCI, USA). Quality control and further analyses were performed using BrB-ArrayTools. RNA degradation plots and 3′ to 5′ signal ratios for probe sets corresponding to *ACTB* and *GAPDH* mRNAs were calculated for quality control assessments. Class comparison tool, which uses random variance model of *t* test for small size samples, was used to find differentially expressed genes between treatment groups. Functional annotations were done using “Functional Annotation Chart” tool of DAVID Bioinformatics Resources 6.7 (NIH, USA).

### CDNA synthesis and quantitative RT-PCR

cDNA was synthesized from 1 μg total RNA, using oligo-dT primers and RevertAid First Strand cDNA Synthesis Kit (Thermo Scientific, K1622) according to user’s protocol. Specific primers for PCR amplification (available upon request) were designed using PrimerBlast of NCBI [80]. qRT-PCR experiments were performed in duplicates, using DyNAmo™ HS SYBR® Green qPCR Kit (Thermo Scientific, F-410 L). *GAPDH* as a reference gene was tested by calculating the significance of the log_2_ fold change of multiple probe sets represented in the array. Our results suggested that *GAPDH* was a reliable and stable reference gene in response to AZA treatment in MCF7, MDA-231, and MCF12A cell lines since no probe set exhibited fold change greater than 1.2 fold (log_2_ fold change: −0.28, FDR >0.1). Thus, *GAPDH* was used as an internal control gene in cell lines, and *ACTB* and *SDHA* geometric means were used [81] for internal control genes in tissue samples. Delta Ct method [82] was used for relative quantification of mRNAs, using the calculated efficiency values of each primer pair. Statistical significance of differences between groups was tested with Mann–Whitney test or Wilcoxon signed rank test (for paired data), using the log_2_ transformed expression values, using GraphPad Prism 6.0 (GraphPad Software Inc., USA).

### DNA isolation, bisulfite treatment and sequencing

DNA was obtained using Nucleospin Tissue kit (Macherey-Nagel, 740952.5) as described in the user protocol. Bilsulfite treatment was carried out with 1 μg DNA, using Epitect Bisulfite Kit (Qiagen, 59104). CpG islands were detected and bisulfite sequencing primers for *TAGLN* gene were designed using the Methyl Primer Express® software (Applied Biosystems, USA). The primers amplified the region between −290 to +117 bp with respect to TSS (GenBank: NM_003186.3) (Left primer: 5′-GGGGTTAGAGAATAGTGAAGTAGGAGTA-3′; Right primer: 5′-ACACTCACAAAACTTCCTCAAAACT-3′). Gel extraction was carried out with the QIAquick Gel extraction kit (Qiagen, 28704). Specific PCR products were cloned into pGEM-T-easy cloning vector (Promega, A1360) after which plasmid isolation of selected colonies (5 for each sample) was performed with the PureLink Quick Plasmid Miniprep Kit (Invitrogen, K210011) and sent for sequencing. Sequencing of the bisulfite-treated DNA inserts was performed with SP6 primers using the dideoxy chain termination method (by Iontek, Istanbul, Turkey). Bisulfite sequencing results were analyzed using QUMA software [83]. Statistical differences between breast cancer and NTB cell lines were tested with Mann–Whitney test. Statistical differences between methylation percentages of paired tissues were tested with Wilcoxon signed rank test, in GraphPad Prism 6.0.

### Breast tumors and normal tissue samples

*TAGLN* expression in publicly available datasets was analyzed using Oncomine™ (Compendia Bioscience, USA). Primary breast tumors and their matched normal tissues (Table 1) were surgically removed from patients (*n* = 21), at Numune Training and Research Hospital, Ankara, Turkey. The use of the tissue material in this project was approved by the Ethics Committee and consents were obtained in accordance with the Helsinki Declaration of 1975. Tumor samples used in the study were composed >90 % of tumor cells, according to hematoxylin and eosin staining. RNA isolation from tissues was performed with Trizol (Life Technologies, 15596–026). Breast tumor cDNA array (OriGene, BCRT101) was composed of 41 tumor and 7 normal tissues. Breast tumor tissue array (BioChain, Z7020005) was composed of 7 non-tumor and 68 tumor tissues of breast.

### IHC staining and scoring

Immunohistochemical studies were performed automatically in the Bond Max equipment (Leica Microsystems Inc.). Antigen retrieval steps were performed in Bond-Epitope Retrieval Solution 1 (Leica Microsystems, Germany) with TAGLN antibody (1:500, Abcam ab1416) at 100 °C. Detection was carried out with Bond Polymer Refine Red Detection kit (Leica Microsystems, DS9390). Stained slides were dehydrated and covered with mounting medium (Dako, CS70330) and cover-slips. Digital images of the slides were evaluated using the H-score method [84]. Statistical differences between groups were calculated with Mann–Whitney test while correlations were tested with Spearman correlation, in GraphPad Prism 6.0.

### Survival analysis and analysis of publicly available methylation data

Survival analyses regarding the expression of *TAGLN* were performed using Kaplan-Meier (KM) plotter, breast cancer cohort [43]. Gene expression information of 205547_s_at probe set corresponding to *TAGLN* was used. Auto cutoff was used to generate the best performing KM curves. To perform methylation analyses, all the methylation data published under super-series GSE20713 (GSE20712 and GSE22249) and a separate dataset (GSE31979), whose data distributions were similar, were downloaded from GEO2R. Both of the studies were performed on GPL8490 Illumina HumanMethylation27 BeadChip arrays. One probe (cg24619694) corresponded to a CpG at +445 position from TSS of *TAGLN*, and the other probe (cg06950730) corresponded to a CpG at −561 position. Methylation scores of two probes were correlated (Spearman coefficient = 0.714, *P* < 0.0001). Thus, average methylation scores of the two probes were used for the calculations. The data were normalized by assigning 0 for the lowest average methylation scores for two probes of *TAGLN* gene, and assigning the value 100 for the highest scores for each dataset, and the scores in between are fit into a sigmoidal curve. Normalized methylation scores (NAMS) were used for the further studies. Mann–Whitney test was used to compare two groups. For correlation analyses, expression data (GSE20711) corresponding to 88 breast cancer patients and 2 healthy individuals included in the methylation study (GSE20713) was downloaded from GEO2R, and the Spearman’s rank correlation between expression and methylation scores was calculated. For survival analyses using the NAMS values, a range of values greater than or equal to the diagnostic cutoff (i.e., 60, 65, 70, 75, 80, 85, and 90) was tested, and the best performing cutoff was determined to be NAMS >80 [85]. Patients were divided into NAMS ≥80 and NAMS <80 groups, and 10-year RFS were calculated for each group. *P* values were calculated using Gehan-Breslow-Wilocoxon test in GraphPad Prism 6.0.

### Western blotting

Protein lysates (20–50 mg) from cell lines were separated under reducing conditions (5 % β-mercaptoethanol), in 12 % Tris-glycine gels and SDS-Tris-glycine running buffer and were blotted on nitrocellulose membranes. Membranes were incubated overnight at 4 °C, with primary antibodies (α-TAGLN, Abcam ab1416, 1:2,000; α-Calnexin, Abcam, 1:20,000), followed by washing with Tris buffered saline –Tween-20 (0.25 %, TBS-T) and secondary antibody (horse radish peroxidase-conjugated anti-rabbit IgG, Abcam 1:5,000) incubations for 1 h at room temperature. Signal detection was performed with ECL prime system (Amersham Life Science, RPN2232).

### siRNA transfections and overexpression of *TAGLN* in breast cancer cells

siGENOME Human *TAGLN* siRNA (SMARTpool, Thermo Scientific, M-003714-02-0020) and siGENOME Non-Targeting siRNA #2 (Thermo Scientific, D-001210-02-20) control siRNA transfections were performed with RNAi-max (Invitrogen, 13778150), using the reverse transcription protocol supplied by the producer. *TAGLN* overexpression, TrueORF-Gold pCMV6-Entry-*TAGLN* (Origene, RC215789), and control pCMV6-Entry vectors (Origene, PS100001) were transfected to MDA-MB-361 and MDA-MB-157 cells using Lipofectamine 2000 reagent (Invitrogen, 11668019). Stable clones were generated with Geneticin (Gibco-Life Technologies, 10131–035) selection.

### 2D colony formation assays

Cells were seeded to be 2.000/well in 6-well plates and were grown for 2 weeks (MDA-MB-157, MCF10A, and MCF12A) or for 4 weeks (MDA-MB-361). Colonies were then stained with 0.5 % crystal violet and counted using Image J (NIH). Colony sizes of MDA-MB-361 were calculated using Image J. Colony sizes of other cell lines could not be determined due to more scattered nature of colonies of these mesenchymal types of cells. Statistical differences were calculated with two sample *t* test in GraphPad Prism 6.0.

### *In vitro* wound healing and Matrigel invasion assays

For *in vitro* wound healing assays, cells were plated to be confluent in 24-well plates. Next day, media was changed to low serum media (0.5 % FBS) and at least 6 independent scratches/sample were made with 200-μl pipette tips, and photos were taken after 24 h. Statistical differences between groups were tested with the Mann–Whitney test in GraphPad Prism 6.0. For Matrigel invasion assays, Growth Factor Reduced Matrigel Matrix Basement Membrane (BD, 356230) was diluted (1:5) and added to Transwell Permeable Support invasion chambers (Corning, 3422) in 24 wells to form a thin layer. Cells (150,000) per chamber were added to the upper chamber with 0.5 % serum containing media and the lower chamber was supplied with 10 % serum containing medium. Cells on the lower side of the chamber were stained with Giemsa and counted at the 48^th^ hour.

## Availability of supporting data

The data set supporting the results of this article is available in the Gene Expression Omnibus (GEO) repository, [GSE71363, http://www.ncbi.nlm.nih.gov/geo/query/acc.cgi?acc=GSE71363].

## Additional files

Additional file 1:
**Expression changes upon AZA treatments.** Probe IDs, gene names, and fold change ratios of the genes altered upon AZA treatment in each cell line at 1.5 fold and *P* < 0.05 have been listed. (XLSX 2886 kb) Additional file 2:
**Expression in untreated breast cancer cell lines in comparison to MCF12A.** Probe IDs, gene names, and relative expressions in breast cancer cell lines with respect to untreated MCF12A at 1.5 fold and *P* < 0.05 have been listed. (XLSX 11397 kb) Additional file 3:
**Final gene lists altered upon AZA treatments only in breast cancer cell lines.** Probe IDs, gene names, accession numbers and fold change ratios of the genes altered upon AZA treatment, in each breast cancer cell line at 1.5 fold and *P* < 0.05, and filtered as shown in Fig. 1A have been listed. (XLSX 387 kb) Additional file 4:
**Tables not included in the main manuscript have been listed.** **Table S1.** Number of probe sets affected by AZA treatment; **Table S2.** Comparison of significantly altered probe sets with the independent study GSE20713 Dataset; **Table S3.** Cancer vs. normal analysis of *TAGLN* mRNA in Oncomine database. (PDF 18 kb) Additional file 5: Figure S1.
*TAGLN* expression and methylation status, and lymph node metastasis of breast cancer; **Figure S2.** Univariate survival analysis of *TAGLN* microarray expression levels; **Figure S3.** Effect of *TAGLN* over-expression on migration and invasion of MDA-MB-157 cells. (PDF 1151 kb)

## Abbreviations

ANLN: Anillin
ARE: average relative expression
AZA: 5-aza-2′-deoxycytidine, Decitabine
BC: breast carcinoma
DMSO: dimethyl sulfoxide
LN: lymph node
NAMS: normalized average methylation score
NTB: non-tumorigenic breast
ROC: receiver-operator characteristics
SEM: standard error of means
TAGLN: transgelin
TN: triple negative
TSG: tumor suppressor gene
