# Supplementary material for: Transgelin gene is frequently downregulated by promoter DNA hypermethylation in breast cancer
Source: Clin Epigenetics. 2015 Sep 28;7:104. doi: 10.1186/s13148-015-0138-5 (PMC4587865; doi:10.1186/s13148-015-0138-5)
Supplement: Additional file 4: — Tables not included in the main manuscript have been listed. Table S1. Number of probe sets affected by AZA treatment; Table S2. Comparison of significantly altered probe sets with the independent study GSE20713 Dataset; Table S3. Cancer vs. normal analysis of TAGLN mRNA in Oncomine database. (PDF 18 kb) [file 13148_2015_138_MOESM4_ESM.pdf]

Supplementary Tables

Table S1. Number of probe sets affected by AZA treatment

	MDA-MB-231		MCF7		MCF-12A	
***P* values**	**UP**	**DOWN**	**UP**	**DOWN**	**UP**	**DOWN**
***P* < 0.05**	4284	3679	3993	4634	2981	2321
***P* < 0.01**	4253	3654	3903	4531	2868	2074
***P* < 0.001**	3832	3282	3044	3061	2081	1309
***P* < 0.0001**	2346	1823	1896	1329	985	389

*Numbers of probe sets under given *P* values were determined with class comparison tool of BRB-ArrayTools, which uses random variance model of unpaired t-test. 1.5-fold threshold was used.

Table S2. Comparison of significantly altered probe sets with the independent study GSE20713 Dataset

Gene lists	Total [a]	Significantly Altered in GSE 20713[b]
**MCF7 UP**	1145	431 (37.6%)
**MCF7 DOWN**	1876	799 (42.6%)
**MDA-MB-231 UP**	913	250 (27.4%)
**MDA-MB-231 DOWN**	1447	533 (36.8%)
**COMMON UP**	198	106 (53.5%)
**COMMON DOWN**	262	180 (68.7%)

[a] Total number of up/downregulated probe sets by AZA in indicated cell lines, but not in MCF12A, by 1.5-fold, at $P < 0.05$ in our set up. [b] Number (and percentage) of up/downregulated probe sets also in GSE20713 dataset, $P < 0.05$, by paired t-test.

Table S3. Cancer vs. normal analysis of *TAGLN* mRNA in Oncomine database

**Study**	**Cancerous tissue being compared to Normal**	***P* value**	**Fold Change**	**Gene Rank (Top % genes)**
Richardson Breast 2 (n = 47)	Ductal Breast Carcinoma	1.03E-12	-5.92	0.3
Turashvili Breast (n = 30)	Invasive Lobular Breast Carcinoma	8.20E-04	-2.26	0.4
	Invasive Ductal Breast Carcinoma	1.50E-02	-2.95	4.9
Ma Breast 4 (n = 66)	Ductal Breast Carcinoma in Situ Epithelia	2.90E-07	-2.78	0.4
	Invasive Ductal Breast Carcinoma Epithelia	4.01E-05	-2.28	1.2
TCGA Breast (n = 593)	Mucinous Breast Carcinoma	9.64E-07	-2.37	1.2
	Invasive Ductal Breast Carcinoma	3.48E-38	-2.70	1.5
	Invasive Breast Carcinoma	1.69E-19	-2.32	3.1
	Mixed Lobular and Ductal Breast Carcinoma	8.86E-06	-2.35	3.2
	Intraductal Cribriform Breast Adenocarcinoma	4.10E-02	-3.10	21
	Invasive Ductal and Lobular Carcinoma	4.10E-02	-2.55	23.9
Sorlie Breast (n = 85)	Lobular Breast Carcinoma	3.80E-02	-2.15	6.8
	Ductal Breast Carcinoma	3.00E-02	-2.13	15.4
Perou Breast (n = 65)	Ductal Breast Carcinoma	1.10E-02	-2.10	8.6
Curtis Breast (n = 2 136)	Invasive Ductal Breast Carcinoma	7.15E-50	-2.30	4.7
	Medullary Breast Carcinoma	1.29E-09	-3.37	7.3
	Mucinous Breast Carcinoma	9.39E-14	-2.59	7.5
	Invasive Lobular Breast Carcinoma	2.05E-21	-2.06	7.8
	Breast Carcinoma	5.77E-05	-2.68	8.5
	Invasive Breast Carcinoma	1.68E-04	-2.62	12.0
Finak Breast (n = 59)	Invasive Breast Carcinoma Stroma	2.65E-05	-3.84	33.4
