# Supplementary material for: Transgelin gene is frequently downregulated by promoter DNA hypermethylation in breast cancer
Source: Clin Epigenetics. 2015 Sep 28;7:104. doi: 10.1186/s13148-015-0138-5 (PMC4587865; doi:10.1186/s13148-015-0138-5)
Supplement: Additional file 5: Figure S1. — TAGLN expression and methylation status, and lymph node metastasis of breast cancer; Figure S2. Univariate survival analysis of TAGLN microarray expression levels; Figure S3. Effect of TAGLN over-expression on migration and invasion of MDA-MB-157 cells. (PDF 1151 kb) [file 13148_2015_138_MOESM5_ESM.pdf]

Supplementary Figures

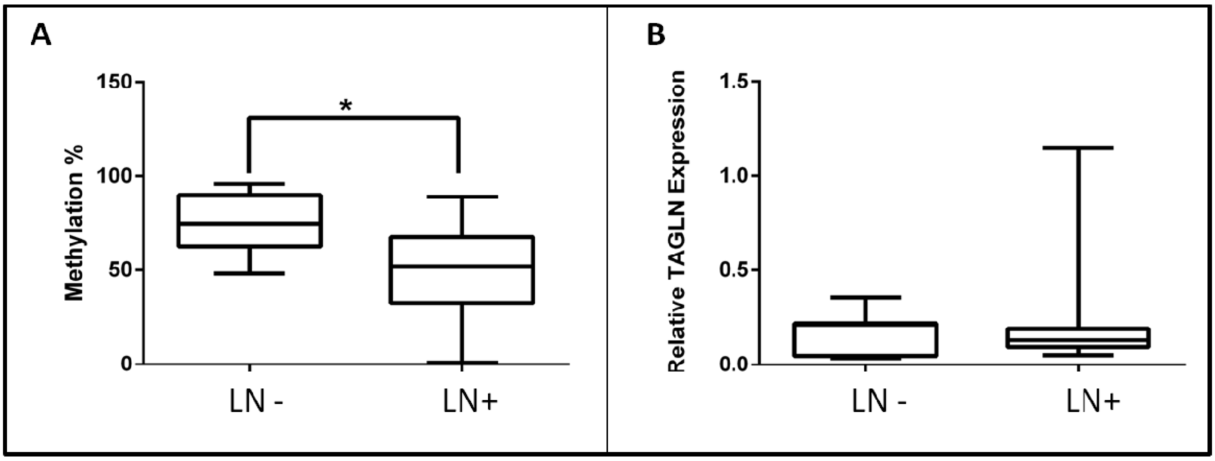

Figure S1. ***TAGLN*** **expression and methylation status, and lymph node metastasis of breast cancer (A)** Methylation levels of *TAGLN* were significantly higher in lymph node negative (LN-) tumors (*$P < 0.05$, Mann Whitney Test), **(B)** while mRNA expressions did not differ. The geometric means of *ACTB* and *SDHA* expressions were used as reference genes in qRT- PCRs analysis. Horizontal lines show maximum, median and minimum values.

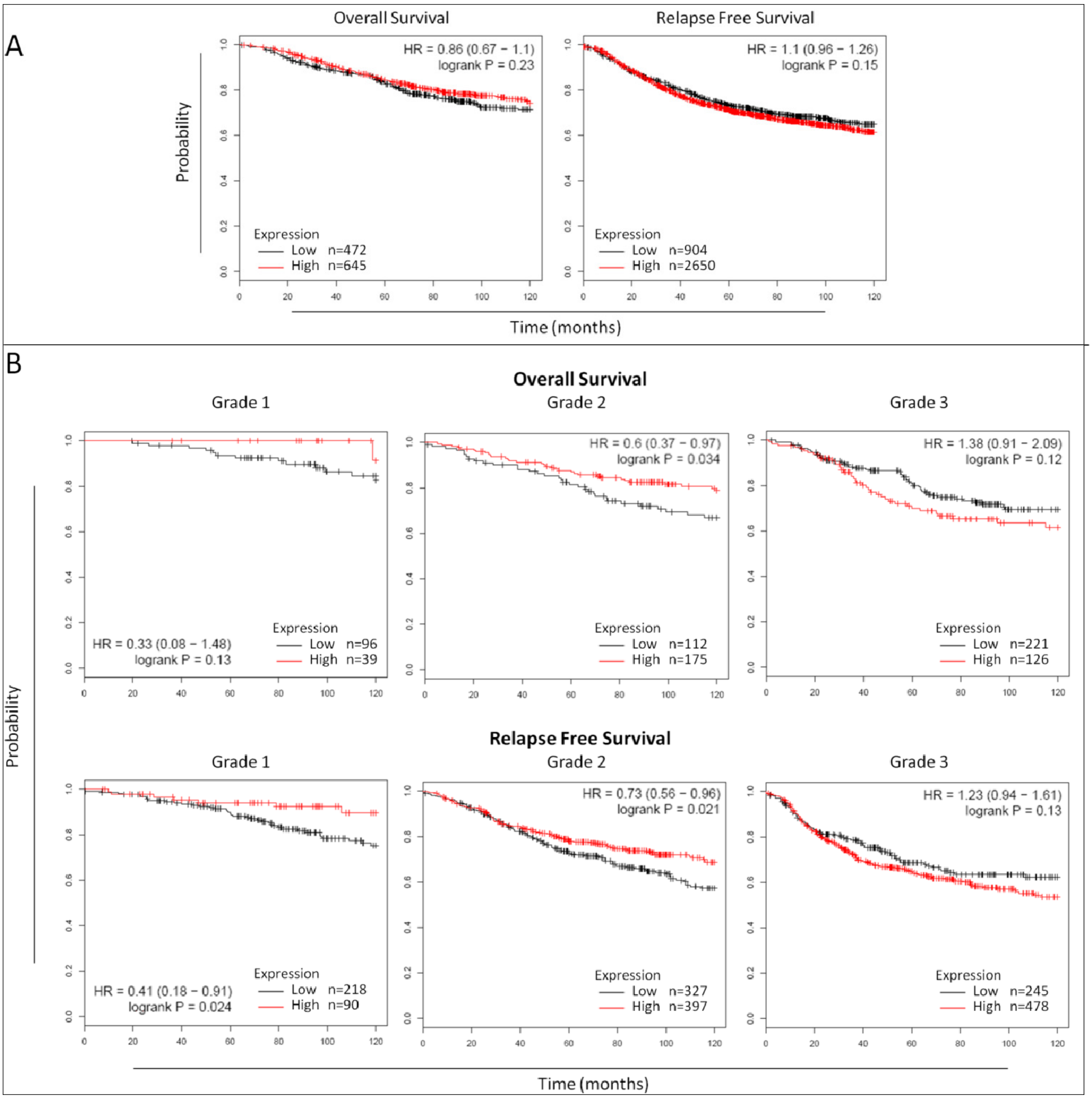

Figure S2: Univariate survival analysis of *TAGLN* microarray expression levels. KM plots were generated for *TAGLN* (205547_s_at) expression using KM plotter breast cancer cohort (http://www.kmplot.com/). **(A)** *TAGLN* expression does not affect either OS or RFS when all patients are analyzed together. **(B)** Higher expression of *TAGLN* results in significantly better OS in grade 2 patients, and RFS in grade 1 and grade 2 patients, while there was no significant difference in grade 3 patients.

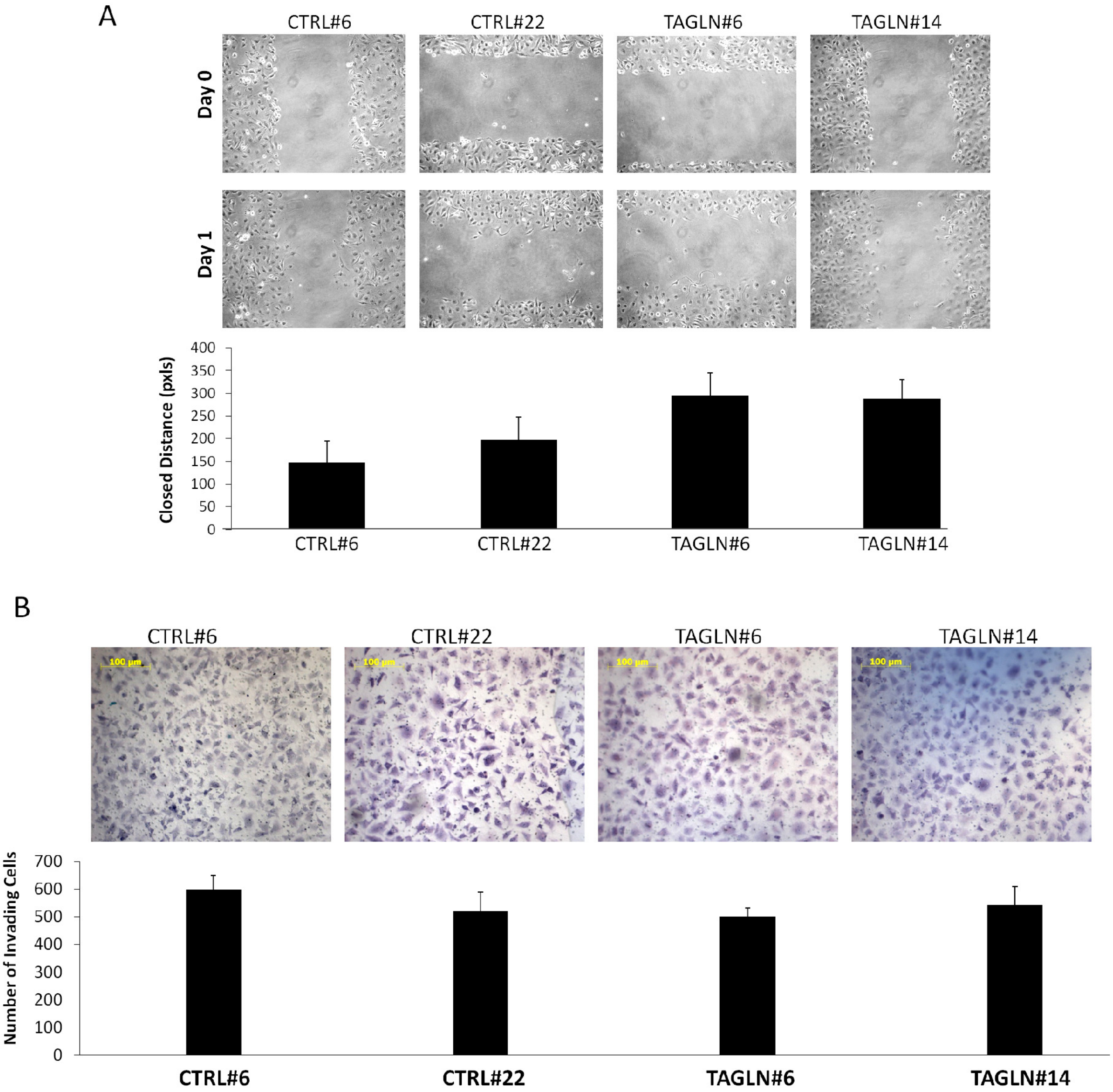

Figure S3: Effect of *TAGLN* over-expression on migration and invasion of MDA-MB-157 cells. *TAGLN* overexpression in MDA-MB-157 cells did not result in a significant difference in either migration (A), or invasion (B) of normally motile MDA-MB-157 cells. CTRL#6 and CTRL#22: MDA-MB-157 cells transfected with control vector. TAGLN#6 and TAGLN#14 : MDA-MB-157 clones transfected with *TAGLN* over-expression vector.
